# Modulation of the rumen microbiome and metabolism in dairy cows by altering the concentrate feeding pattern and the inclusion of *Saccharomyces cerevisiae* yeast

**DOI:** 10.3389/frmbi.2026.1884444

**Published:** 2026-08-07

**Authors:** Timothy J. Snelling, Catherine A. Johnson, Helen E. Warren, Jules Taylor-Pickard, James A. Huntington, Liam A. Sinclair

**Affiliations:** 1 Animal Science Research Centre, Harper Adams University, Edgmond, United Kingdom; 2 Alltech Bioscience Centre, Dunboyne, Ireland

**Keywords:** concentrates, diet selection, microbiome, pH, rumen, volatile fatty acids

## Abstract

Dairy cattle are typically fed a total mixed ration (TMR), which is prepared in an automated mixer wagon. On-farm, effective TMR mixing can often be neglected due to lack of time or training. This leads to a disbalance of intake and potentially detrimental effects on health and production. Using dietary treatments to simulate this effect, this study determined the response of rumen metabolism and microbiome to different concentrate allocations in combination with a live *Saccharomyces cerevisiae* supplement (yeast supplementation, YS). The 4 × 4 Latin square design consisted of four dairy cows fitted with permanent rumen cannulae, which were fed a partial mixed ration with dietary concentrates (4 kg per cow per day) in an even or an uneven pattern of allocation (concentrate allocation, CA). YS was included in the TMR at a rate of 1 g per cow per day. Rumen metabolism was determined by measuring the pH, volatile fatty acids (VFAs), and ammonia nitrogen (NH_3_–N). The rumen microbial community was characterised using 16S rRNA gene amplicon sequencing. Both CA and YS had no effect (*p* > 0.05) on the dry matter intake, milk yield, or composition. CA did not affect the rumen NH_3_–N and VFA concentrations (*p* > 0.05). YS inclusion tended to increase the rumen pH (*p* = 0.088), acetate (*p* = 0.076), and valerate (*p* = 0.091). YS significantly increased the total VFA (*p* = 0.033) and propionate concentrations (*p* < 0.016). CA had little overall effect on the rumen microbiome beta diversity. However, there was a reduction in the relative abundance of a Prevotellaceae feature associated with an uneven pattern of CA. Bray–Curtis clustering of the microbiome was observed with YS (*p* = 0.002), driven by a decrease of Gammaproteobacteria and Prevotellaceae features and an increase of a Christensenellaceae feature (LDA > 2.0).

## Introduction

Total mixed rations (TMRs) are a popular feeding system for dairy livestock as they provide a diet that is formulated to meet the energy requirements to maintain optimum production and health, with the concentrate, forage, and supplement components combined and dispensed via a mixer wagon (Schingoethe, 2017). Several studies have been undertaken to compare the effect of feeding concentrates as part of a TMR or separately, either at milking or via out-of-parlour feeders (Little et al., 2016, 2018; Purcell et al., 2016). However, if the TMR is poorly mixed, then cattle are more likely to select the more palatable concentrates, which are rapidly fermented in the rumen (Tayyab et al., 2018), leading to an imbalance in the dietary nutrient intake. This can affect the composition of the rumen microbiome and the production of microbial fermentation products, which can chronically reduce the rumen pH, affecting health and performance (Jin et al., 2024; Belanche et al., 2012; Zhang et al., 2020). In a recent survey of feeding practice on UK dairy farms, 58% had poorly mixed rations, with 66% having evidence of diet selection at 4 h post-feeding (Tayyab et al., 2018). This combination of poorly mixed rations and diet selection can increase the risk of sub-acute ruminal acidosis (SARA) and decreased performance (Agle et al., 2010). SARA is normally associated with an increase in the concentration of volatile fatty acids (VFAs) (Pourazad et al., 2016), a decrease in the abundance of fibrolytic bacteria such as Ruminococcaceae, and an increase in lactate-producing bacteria such as *Streptococcus bovis* and *Lactobacillus* spp (Petri et al., 2017, 2013; Zhang et al., 2020).

Supplements are sometimes included in the diets of livestock animals to ameliorate some of the harmful effects of dietary imbalances by preventing dysbiosis of the rumen microbiome and associated pathologies (Abdelli et al., 2022; AlZahal et al., 2014; Johnson et al., 2023). Specifically, yeast supplementation (YS) has been reported to promote fibre digestion by promoting the growth of fibre-degrading rumen microbes such as *Fibrobacter* and *Ruminococcus flavefaciens* (Chaucheyras-Durand et al., 2016; Newbold et al., 1996). Yeast supplements have also been reported to reduce the variability of rumen pH by oxygen scavenging and the reduction of lactate through redirecting substrate availability away from lactate production (Chaucheyras-Durand et al., 2008).

Despite the prevalence of diet selection on dairy farms, only a few studies have been undertaken to determine its impact on the rumen microbiome and metabolism or the effectiveness of supplements to ameliorate the potential negative effects. Therefore, this study provides novel insights by deliberately encouraging diet selection through manipulating the composition of the diet at feedout and by measuring the effects on intake, performance, and health. The formulation of the ration was not intended to induce acidotic conditions in the rumen for ethical reasons. However, altering the concentrate allocation (CA) was designed to shift the rumen conditions without harming the experimental animals to allow inference of potential pathologies in more extreme nutritional models. The addition of live yeast (*Saccharomyces cerevisiae*) would determine whether the supplement would play a part in countering any potential harmful effects. Measurement of the rumen metabolism and the microbiome provides evidence of associations between the diet and the microbial composition and activity that may underpin the effects of the diet on dairy cow.

## Materials and methods

### Animals, diets, and management

Four second-lactation Holstein–Friesian dairy cows that were 69 ± 12.1 days (mean ± SE) in milk, weighing 650 ± 26.2 kg and yielding 40 ± 2.6 kg milk per day, and had been fitted with permanent rumen cannula (10 cm diameter; Bar Diamond Inc., Parma, ID, USA) at the end of their previous lactation were used. The study used a 4 × 4 Latin square design with four periods, each of 35-day duration, with 28 days for adaptation to the diets and sampling during the final 7 days of each period. Carryover effects were not specifically tested for or controlled. However, the 4 × 4 Latin square was selected from a randomised design to reduce the carryover effects by eliminating the same treatments following in successive periods. The cows were housed loose in an indoor pen that was bedded with sawdust and manually scraped daily. Access to the diets was provided via individual feed bins (American Calan Inc., Northwood, NH, USA). During the sampling period, the cows were restrained in individual tied stalls fitted with 15-cm-deep foam mattresses. Throughout the study, the cows were milked twice daily at 0600 and 1600 hours (Milkline, London, UK), and free access to water was provided at all times. All cows received a partial mixed ration (PMR) containing a pelleted concentrate, grass, and maize silages at a ratio [in kilograms per kilogram, dry matter (DM) basis] of 0.297, 0.318, and 0.385, respectively, which was fed fresh daily at 0800 hours at a rate of 110% of the previous days’ intake (**Table 1**). The concentrates were prepared with (+) or without (−) live *S. cerevisiae* yeast (YS) (Yea Sacc^®^, Alltech, Nicholasville, KY, USA; contents: 1 × 10^9^ colony forming units per gram product) to provide the manufacturer’s recommended rate of 1 g per cow per day. To simulate the effect of diet selection by the cow, an additional 4 kg per cow per day of the concentrates was added to the PMR either in an uneven pattern of allocation (U) (4 kg at 0600 hours) or an even pattern of allocation (E) at 0600, 1000, 1400, and 1700 hours (1 kg at each time point). This provided four treatments: U−, uneven pattern of concentrate without yeast; U+, uneven pattern of concentrate with yeast; E−, even pattern of CA without yeast; and E+, even pattern of CA with yeast.

**Table 1:** Intake and performance of dairy cows receiving an even (E) or an uneven (U) pattern of concentrate allocation (CA) either with (+) or without (−) *Saccharomyces cerevisiae* inclusion (YS).

Cow production	Treatment				SED	*p*-value		
	E+	E−	U+	U−		CA	YS	CA × YS
Total DM intake (kg/day)	23.2	22.6	22.9	23.6	0.48	0.367	0.943	0.103
Performance								
Milk yield (kg/day)	39.0	40.0	39.1	38.8	1.30	0.560	0.717	0.512
Milk fat (g/kg)	38.2	41.2	39.1	40.9	5.43	0.933	0.552	0.876
Milk fat (kg/day)	1.65	1.59	1.65	1.57	0.123	0.880	0.453	0.922
Milk protein (g/kg)	27.8	30.1	27.8	30.6	2.65	0.871	0.219	0.904
Milk protein (kg/day)	1.20	1.21	1.18	1.18	0.041	0.388	0.895	0.810
Milk urea–N (g/kg)	0.26	0.25	0.25	0.25	0.002	0.706	0.578	0.920
Live weight (kg)	693	689	700	705	10.4	0.154	0.948	0.564
Live weight change (kg/day)	0.26	0.36	1.06	0.53	0.401	0.138	0.479	0.331
Body condition score	2.38	2.44	2.44	2.56	0.084	0.168	0.168	0.620
Whole tract digestibility (kg/kg)								
DM	0.710	0.705	0.715	0.711	0.0048	0.156	0.247	0.943
OM	0.736	0.731	0.738	0.736	0.0050	0.365	0.418	0.702
NDF	0.673	0.660	0.653	0.688	0.0151	0.723	0.383	0.089
Blood metabolites (mmol/L)^a^								
NH_3_	43.3	44.1	39.2	44.1	1.83	0.165	0.072	0.170
Glucose	3.89	3.75	3.76	3.79	0.240	0.812	0.748	0.653
BHB	0.65	0.61	0.74	0.76	0.102	0.161	0.920	0.694
Urea	3.34	2.97	3.518	3.27	0.301	0.295	0.199	0.773

CA, main effect of pattern of concentrate allocation; YS, main effect of the inclusion of yeast; CA × YS, interaction between the pattern of concentrate allocation and the inclusion of yeast; DM, dry matter; OM, organic matter; NDF, neutral detergent fibre; BHB, β-hydroxybutyrate.

^a^ Mean of the blood samples taken at 0600, 1000, 1400, and 1700 hours.

**Table:** 

Cow	Treatment period			
	1	2	3	4
1	C	A	B	D
2	A	D	C	B
3	B	C	D	A
4	D	B	A	C

A: U−, 4 kg concentrates fed in the first meal, no supplement.

B: U+, 4 kg concentrates fed in the first meal, with YS.

C: E−, 4 kg concentrates in four meals, no supplement.

D: E+, 4 kg concentrates in four meals, with YS.

During the sampling days, refusals were collected daily to calculate the intake, with subsamples of forages, PMR, and concentrate also collected and stored at −20°C for subsequent bulking and analysis. Milk was collected on three separate days during the sampling period at both the morning and afternoon milking times (0600 and 1600 hours) for subsequent analysis by National Milk Laboratories (NML; Wolverhampton, UK) using near mid-infrared (MIR; Foss, Hillerød, Denmark). The total faecal output was collected over a 5-day period when the cows were restrained in the stalls, with 2.5% of the subsamples stored at −20°C before bulking within cow and period. The body weight (Tru-Test, Auckland, New Zealand) and the condition score (Ferguson et al., 1994) were recorded post-milking at 0700 hours at the start and the end of each period.

### Rumen and blood sampling

Rumen fluid samples were collected at 0600 hours, prior to feedout, and then at 0900, 1200, 1500, 1800, and 2100 hours on day 31 of each period. To obtain a representative sample, four grab samples of digesta were taken via the cannula from within the dorsal region of the reticulorumen by inserting the arm approximately 50 cm down through the cannula, pooled, and strained using four layers of muslin cloth to separate the solid (SPD) and liquid (LPD) phases of the digesta. An aliquot of the liquid phase was immediately analysed for pH (Hanna Instruments, Bedfordshire, UK). A sample of the LPD (45 ml) was added to 5 ml metaphosphoric acid (25% *w*/*v*), mixed, and stored at −20°C prior to VFA and ammonia N analysis. For the 0600-, 0900-, and 1800-h sampling times, a second subsample of the LPD (10 ml) was added to 10 ml of a glycerol solution (30%, *v*/*v*), and a representative sample of the SPD was placed into a 100-ml container and topped up with glycerol solution (15%, *v*/*v*). Both were stored at −20°C prior to DNA extraction for microbial community analysis.

Blood samples were collected by jugular venipuncture at 0600, 1000, 1400, and 1700 hours over two consecutive days into sodium heparin BD Vacutainers^®^, centrifuged at 1,500 × *g* for 10 min, and the plasma extracted and analysed within 30 min for ammonia (Richardson et al., 2003) with the remainder stored at -20° for subsequent analysis.

### Chemical analysis

Samples of the grass and maize silages, PMR, and concentrates, as well as faecal samples, were bulked between days within each sampling week of each period and were analysed according to the Association of Official Analytical Chemists (2012) for DM [943.01; intra-assay coefficient of variation (CV) of 2.37%], crude protein (CP) (990.03; intra-assay CV of 2.48%), and ash (942.05; intra-assay CV of 0.40%), with neutral detergent fibre (NDF; intra-assay CV of 1.36%) determined according to Van Soest et al. (1991) using heat-stable α-amylase (Sigma, Gillingham, UK) and sodium sulphite and expressed exclusive of residual ash. The rumen fluid VFA, lactate, and ammonia–N concentrations were analysed as described by MAFF, (1986) and Johnson et al. (2023). Blood plasma samples were analysed using a Cobas Mira Plus autoanalyser (ABX Diagnostics, Bedfordshire, UK) for glucose, β-hydroxybutyrate (BHB), urea, and ammonia, with intra-assay CVs of 1.72%, 4.28%, 1.78%, and 4.16%, respectively. The kits used were: GLUC-HK (ref GU611), RANBUT (ref RB1008), UREA (ref UR221), and NH3 (ref AM1015), respectively.

### 16S rRNA gene amplicon library preparation

SPD samples were rinsed with 0.9% (*w*/*v*) saline solution and homogenised in a stomacher (Seward, West Sussex, UK) at 230 rpm for 5 min to detach fibre-associated microbes (Ramos et al., 2009). Both the supernatant from this process and the rumen liquid samples (5 ml) were centrifuged for 20 min at 10,000 × *g*, and 0.25 g of the microbial pellet was transferred into a 2-ml screw-top tube for DNA extraction. DNA was extracted following the protocol of Yu and Morrison (2004) by repeated bead-beating followed by precipitation, elution, and purification using columns and reagents from the QIAamp^®^ DNA Stool Mini Kit (Qiagen Ltd., Manchester, UK).

PCR amplification was carried out in triplicate 25 μl reactions using Q5^®^ High-Fidelity DNA polymerase (New England Biolabs Inc., Hitchin, UK) with universal prokaryotic primers targeting the V4 (F515–R806) region of the 16S rRNA gene (read 1: TATGGTAATTGTGTGCCAGCMGCCGCGGTAA; read 2: AGTCAGTCAGCCGGACTACHVGGGTWTCTAAT; index primer: ATTAGAWACCCBDGTAGTCCGGCTGACTGACT) (Thompson et al., 2017), yielding amplicons of approximately 250 nt in length plus barcodes and adapters. Individual samples were identified using eight unique nucleotide barcodes built into both forward and reverse primers (Kozich et al., 2013). The PCR products were cleaned and quantitated using the Quant-It™ PicoGreen™ High-Sensitivity dsDNA Assay Kit (Fisher Scientific UK Ltd., Loughborough, UK). The samples were pooled in equimolar quantities and 80 μl run on a 1% (*w*/*v*) agarose/TBE gel to separate residual primers and dNTPs. The band at the expected size containing the amplicons was excised and purified using a Promega Wizard^®^ SV Gel purification kit (Promega UK, Southampton, UK).

The libraries were quality assessed using an Agilent 2100 Bioanalyzer System (Agilent Technologies, Santa Clara, CA, USA) and sequenced using the Illumina MiSeq v2–250 paired-end reagent kit (Illumina UK, Cambridge, UK) at Edinburgh Genomics (University of Edinburgh, UK). Raw sequence data were uploaded to the European Nucleotide Archive (ENA), with study accession number PRJEB44109 and sample accession numbers ERR10854553–ERR10854660. Sequence data were analysed using QIIME2 (Bolyen et al., 2019) with the DADA2 method, dada2 denoise-single (Callahan et al., 2016), to produce feature tables. As part of the QIIME2 DADA2 pipeline, the q2-dada2 plug-in filtered the phiX reads and chimeras. Sequences were trimmed left at 20 nt and truncated to 220 nt length based on a quality plot.

To achieve the optimal depth of coverage, the sequence counts in each library were normalised by subsampling to 47,000 reads per sample. The SILVA 138 SEED reference database was used for classification of the taxon (phylum) summaries (Yilmaz et al., 2014). Taxonomic classification of the representative sequences of discriminant features was carried out using a BLASTn search against type material from the NCBI reference database (Altschul et al., 1990).

### Statistical analysis

Normality of data was determined using quantile–quantile (*q*–*q*) plots. *A priori* power calculations were carried out using G*Power (Faul et al., 2009). At an effective sample size, including interactions, of the 4 × 4 Latin square, the *α* error probability was 0.05 and the *β* error probability was 0.60.

Data were analysed as a Latin square design using GenStat 18.1 (VSN International Ltd., Oxford, UK), with main effects of CA, addition of yeast (YS), and their interaction using the following model:


$$\text{Y}=\text{μ}+\text{ C}{\text{A}}_{\text{i}}+\text{ Y}{\text{S}}_{\text{j}}+\text{ C}{\text{A}}_{\text{i}}\times \text{Y}{\text{S}}_{\text{ij}}+{\text{P}}_{\text{k}}+{\text{A}}_{\text{l}}+{\text{Ɛ}}_{\text{ijkl}}$$

where *Y* is the observation, *μ* is the overall mean, CA*_i_* is the concentrate pattern effect, YS*_j_* is effect of the addition of yeast, and CA_i_ × YS*_ij_* is the interaction between CA and YS. *P_k_* period and *A_l_* animal were both treated as random effects to improve statistical power by reducing the number of estimated parameters. *Ɛ_ijkl_* is the residual error. Results are reported as treatment means with standard error of the difference (SED), with the level of significance set at *p* < 0.05 and a tendency stated at *p* < 0.10. Pairwise comparisons of the means were conducted using Tukey’s honestly significant difference (HSD) *post-hoc* test. For microbiome data, the depth of coverage per sample was calculated using Good’s statistic (Good, 1953). Microbial community species richness and diversity are summarised using Faith’s phylogenetic diversity (PD) index (Faith, 1992). Beta diversity was calculated using the Bray–Curtis dissimilarity metric, with the resulting distance matrix used to generate principal coordinates analysis (PCoA) plots with significant differences determined using permutational multivariate ANOVA (PERMANOVA) with 1,000 iterations. Taxonomic biomarkers associated with CA and YS were determined using analysis of compositions of microbiomes with bias correction (ANCOM-BC) (Lin and Peddada, 2020) and linear discriminant analysis (LDA) (Segata et al., 2011).

## Results

### Animal performance, digestibility, and blood metabolites

The pattern of allocation of concentrates or the addition of yeast had no effect (*p* > 0.05) on the DM intake, milk yield, milk fat and protein contents, or live weight (**Table 1**). There was also no effect on the apparent whole tract digestibility of DM, organic matter (OM), or NDF. There was a tendency (*p* = 0.072) for lower plasma ammonia in cows when given YS. However, there were no other effects of treatment on plasma glucose, β-hydroxybutyrate, or urea.

### Rumen metabolism

**Figure 1:**
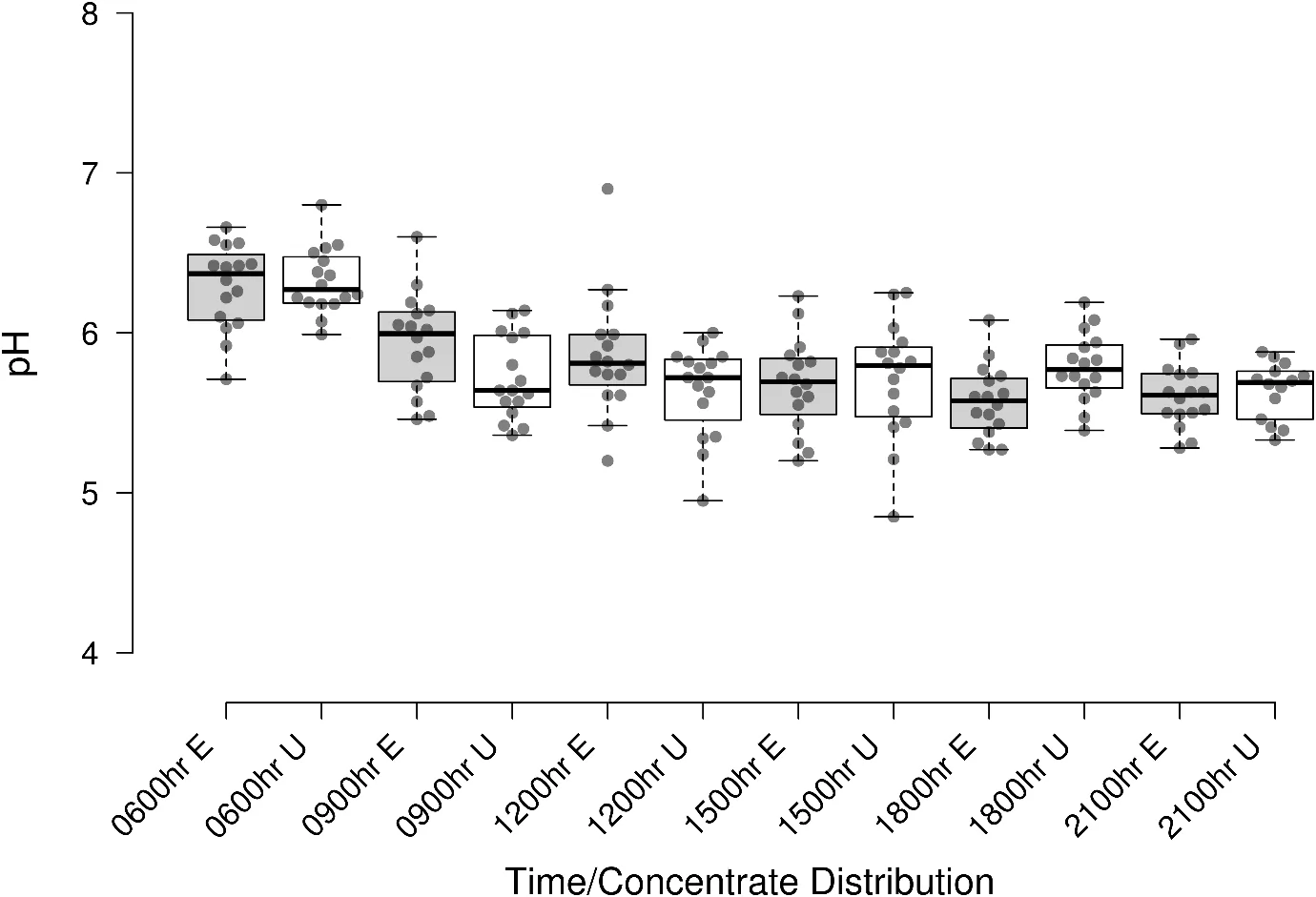
Variability of the rumen pH post-feedout in dairy cows receiving an even (*E*) or an uneven (*U*) pattern of concentrate allocation (CA). *Plotted points* represent individual samples from cows at each time point and period. Interaction between CA and sample time (*p* = 0.018).

The total rumen VFA, butyrate, isobutyrate, valerate, isovalerate, and pH were all affected by sampling time (*p* < 0.05) (**Table 2**). There was no effect on pH with CA (*p* > 0.05). However, there was an interaction between CA and sampling time (*p* = 0.018), with lower pH in the uneven CA samples taken 3–6 h post-feedout (**Figure 1**, **Table 2**). There was a tendency of increased pH with YS (*p* = 0.088), but no interactions found between sampling time and YS. For all sampling times, the minimum mean pH did not fall below 5.6 (**Table 2**).

**Table 2:** Rumen pH, volatile fatty acid (VFA) concentration (in millimolars) by sampling time of day (*T*), and interactions with concentrate allocation (CA)^a^.

Rumen metabolism	Sampling time						*p*-value	
	0600	0900	1200	1500	1800	2100	*T*	*T* × CA
Rumen pH	6.31	5.83	5.75	5.69	5.68	5.62	0.001	0.012
Total VFA	175.5	184.8	174.6	168.9	193.1	153.8	0.041	0.325
Acetate	129.8	129.3	121.2	117.2	134.2	105.6	0.097	0.353
Propionate	25.68	29.90	28.68	27.57	31.67	26.26	0.064	0.781
Butyrate	14.78	18.93	18.19	18.11	20.43	16.44	0.001	0.712
Iso-butyrate	1.094	1.135	1.032	0.904	1.043	0.905	0.001	0.249
Valerate	1.762	2.529	2.494	2.270	2.692	2.090	0.001	0.553
Iso-valerate	2.361	3.030	2.932	2.771	3.066	2.509	0.008	0.558
A/P ratio	5.122	4.352	4.317	4.385	4.337	4.154	0.277	0.799

A/P ratio, acetate/propionate ratio.

^a^ Rumen lactate was measured, but fell below the analytical detection limit (<1 mM) across all dietary treatments and was therefore excluded from the table.

CA had no effect (*p* > 0.05) on the total or individual VFA concentrations. YS increased the total VFA (*p* = 0.033) and propionate concentrations (*p* = 0.016), with the acetate and valerate concentrations tending higher (*p* = 0.076 and *p* = 0.091, respectively) compared with the controls. The proportionate increase in both acetate and propionate meant that no significant change in the acetate-to-propionate (A/P) ratio was found with YS (*p* = 0.517) (**Table 3**). The rumen lactate concentrations were undetectable at the majority of time points, with no differences with CA or YS observed. Supplementation with yeast tended (*p* = 0.100) to increase the rumen NH_3_–N concentration post-feeding.

**Table 3:** Rumen pH, volatile fatty acid (VFA) concentration (in millimolars), and ammonia N concentration (in milligrams per litre) in dairy cows receiving an even (E) or an uneven (U) pattern of concentrate allocation (CA) either with (+) or without (−) *Saccharomyces cerevisiae* inclusion (YS).

Rumen metabolism	Dietary treatment				SED	*p*-value		
	E+	E−	U+	U−		CA	YS	CA × YS
Rumen pH	5.86	5.79	5.84	5.77	0.04	0.592	0.088	0.872
Total VFA	179	168	187	167	7.03	0.620	0.033	0.582
Acetate (A)	125	117	133	118	6.12	0.503	0.076	0.535
Propionate (P)	30.1	27.0	29.6	26.6	1.23	0.703	0.016	0.969
Butyrate	18.2	17.5	18.2	17.4	0.598	0.907	0.189	0.987
Iso-butryrate	1.03	1.04	1.06	1.00	0.032	0.931	0.436	0.322
Valerate	2.39	2.25	2.37	2.22	0.084	0.740	0.091	0.999
Iso-valerate	2.87	2.77	2.87	2.62	0.124	0.574	0.163	0.550
A/P ratio	4.21	4.48	4.52	4.58	0.404	0.404	0.517	0.667
NH_3_–N	65.5	40.4	63.5	52.4	12.4	0.669	0.100	0.391

CA, main effect of pattern of concentrate allocation; YS, main effect of inclusion of yeast; A/P ratio, acetate/propionate ratio.

### Rumen microbiome

**Figure 2:**
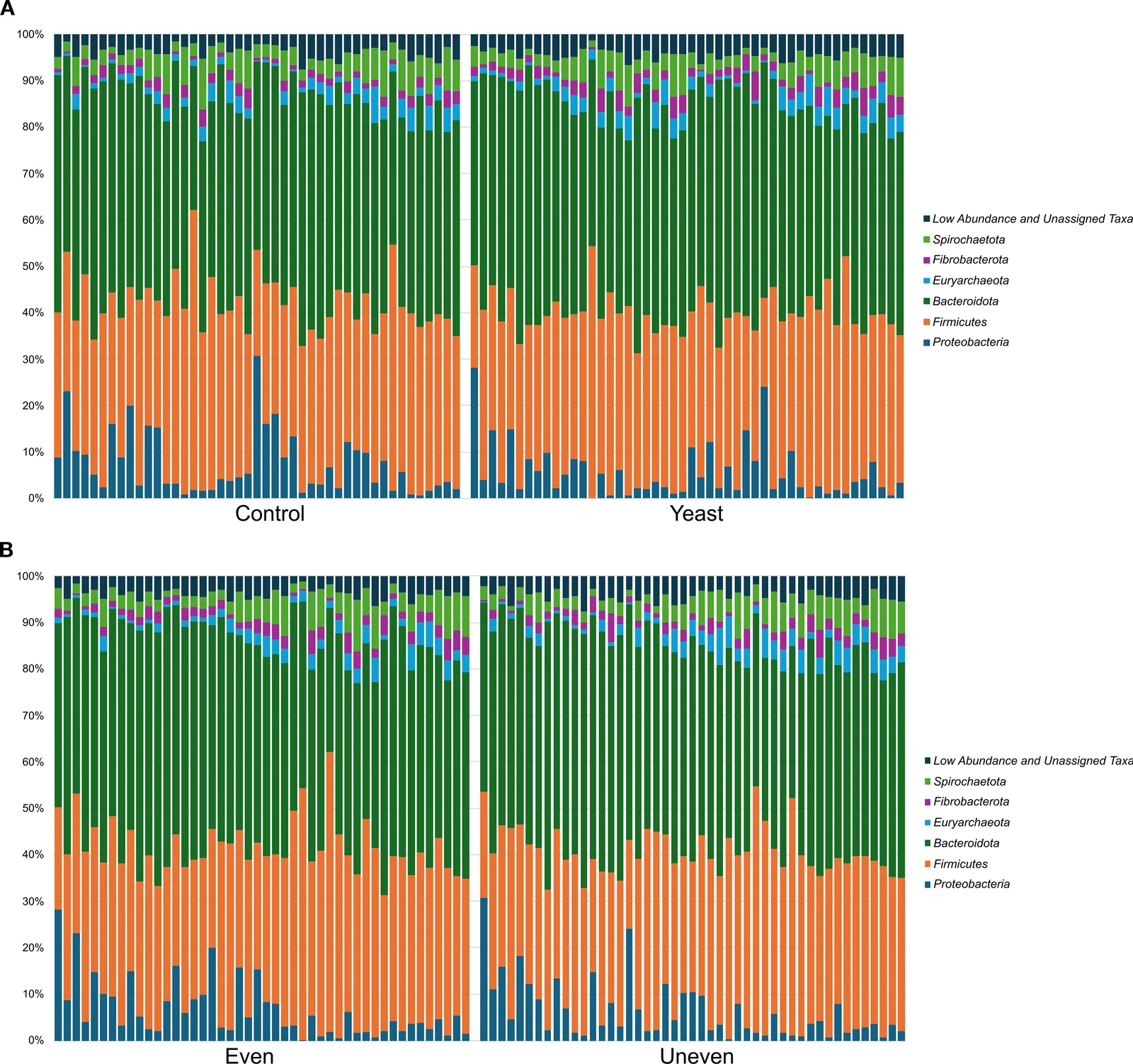
Phylum summaries of the samples based on the concentrate allocation (CA) **(A)** and *Saccharomyces cerevisiae* (YS) inclusion **(B)**. Low abundance and unclassified phyla are compressed into single category.

The datasets generated and analysed during the current study are available in the European Nucleotide Archive repository (https://www.ebi.ac.uk/ena/browser/view/PRJEB44109). Prior to the diversity and statistical analysis, libraries were normalised by subsampling to 47,000 reads per sample. Five libraries from the SPD samples were excluded from the analysis as they lacked sufficient sequence depth: period 1, E− 0600 hours; period 1, E− 0600, 0900, and 1800 hours; and period 4 U− 0600 hours. The relative abundance rates of the taxonomic groups at the phylum level were compared between the CA and YS samples (**Figure 2**). There were no differences found between the alpha diversity (Faith PD, evenness) values by CA or YS (*p* > 0.05).

**Figure 3:**
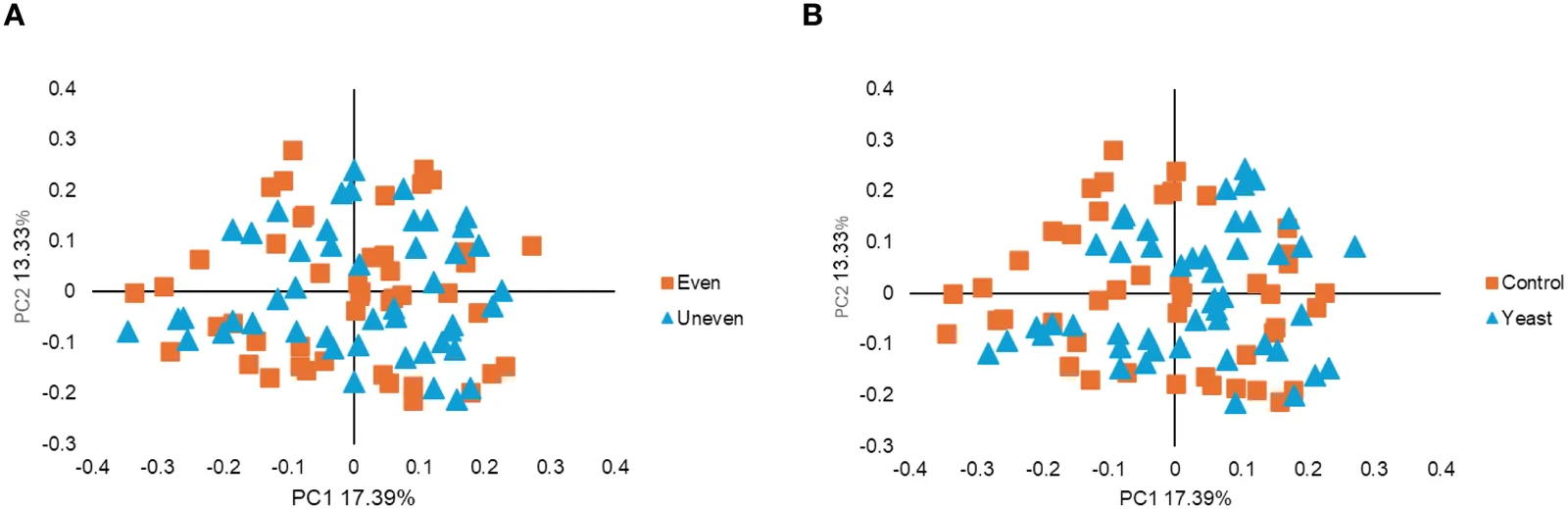
Principal coordinates analysis (PCoA) plot (Bray–Curtis) by concentrate allocation (CA) **(A)** and *Saccharomyces cerevisiae* (YS) inclusion **(B)**. Permutational multivariate ANOVA (PERMANOVA) with 1,000 iterations: *p* = 0.061 (CA) and *p* = 0.002 (YS).

**Figure 4:**
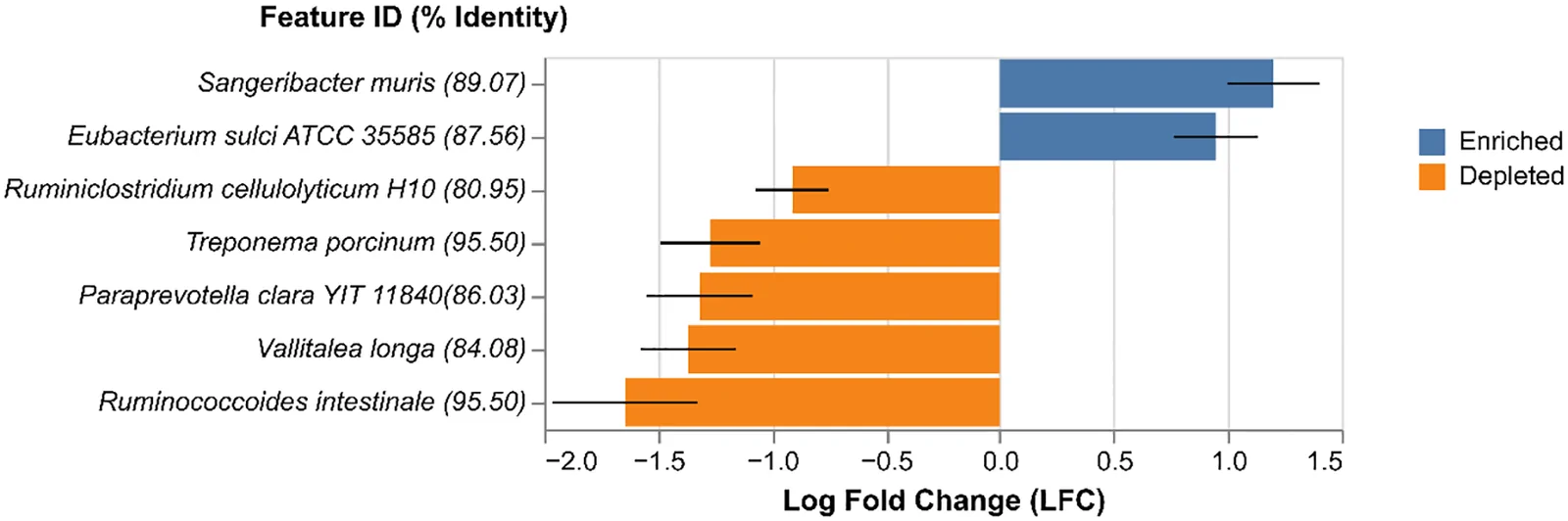
Analysis of compositions of microbiomes with bias correction (ANCOM-BC) of the discriminant features associated with *Saccharomyces cerevisiae* inclusion (YS). Features were identified to the nearest related type strain using BLASTn search of the NCBI GenBank nucleotide database. Note that the classification represents the best culture strain match to the feature representative sequence. Percent ID <95% limits approximation to the genus level or ID <90% to the family level or higher. Taxon IDs are *parallel to the bars* representing enrichment (*blue*) or depletion (*orange*).

The sequence data from the SPD and LPD samples were combined for statistical analysis of the rumen microbiome. PERMANOVA detected microbiome clustering by digesta phase (*p* < 0.001) and YS (*p* = 0.002) and a tendency by CA (*p* = 0.061) (**Figure 3**). ANCOM-BC discriminant analysis did not find any features associated with CA; however, seven features were detected with log fold change associated with YS. Linear discriminant analysis effect size (LEfSe) at false discovery rate (FDR)-corrected values of *p* < 0.05 and LDA scores >2.0 identified one feature associated with CA and three features associated with YS (**Table 4**). Feature identities to the nearest related type strain were determined using a BLASTn search of the NCBI GenBank nucleotide database using the representative sequence of the feature (**Table 4**, **Figure 4**).

**Table 4:** Linear discriminant analysis (LDA) of features based on the mean normalised filtered sequence counts associated with *Saccharomyces cerevisiae* inclusion (YS) and concentrate allocation (CA).

Mean filtered count		*p*-value	LDA	SILVA (% identity)	BLASTn type strain best hit (% identity)
YS−	YS+				
3,719	2,555	0.045	2.77	Gammaproteobacteria unclassified (100)	*Chelonobacter oris* strain 1662 (87)
545.8	242.7	0.009	2.18	Prevotellaceae unclassified (100)	*Prevotella copri* DSM 18205 (90)
514.7	706.0	0.003	2.00	Christensenellaceae R-7 Group (94)	*Gracilibacter thermotolerans* JW/YJL-S1 (87)
					
CA E	CA U				
495.1	283.7	0.010	2.03	Prevotellaceae unclassified (100)	*Prevotella copri* DSM 18205 (90)

Classification represents the best culture strain match to the feature representative sequence. Percent ID <95% limits approximation to the genus level or ID <90% to the family level or higher. FDR-corrected *p* < 0.05, LDA > 2.0.

E, even; U, uneven.

## Discussion

### Animal performance

In high-yielding dairy production systems effective mixing of the TMR is essential to provide a steady supply of nutrients to support production and body condition and to maintain digestive health (Schingoethe, 2017; Tayyab et al., 2018). In the present study, the effect of poor diet mixing and diet selection was simulated by providing different patterns of concentrate allocation relative to time of feed out. It is recognised that this approach does not fully replicate the effect of diet selection on-farm, as cows were fed individually and the daily concentrate allowance kept constant across treatments in the current study. In contrast, diet selection under commercial conditions results not only in a greater rate of intake of rapidly fermentable concentrates post-feeding, but also in a proportion of the cows consuming a greater amount of concentrates, which can impact the rumen microbiome, increase risk of SARA, and affect animal performance (Cheng et al., 2023; Tayyab et al., 2022; Zhang et al., 2020). Saccharomyces cerevisiae yeast’s (YS) mode of action (MOA) is described as oxygen scavenging: production of cofactors (small peptides) that stimulate bacterial growth and sugar-scavenging. Here, the MOA was considered to be directed toward metabolic activity and oxygen scavenging, but replication was considered less of an impact; hence, the additive was fed on a daily basis according to manufacturer’s recommendations.

In the current study, the intake, milk yield, milk composition, diet digestibility, and blood metabolites were not affected either by the pattern of CA or the addition of *S. cerevisiae* (YS). Studies that have investigated the effect of the provision of concentrates in discrete meals in the parlour or via out-of-parlour feeders have also reported small or no significant effects on animal performance, although the response would be influenced by the forage-to-concentrate ratio and the composition of the concentrate (Little et al., 2016, 2018; Purcell et al., 2016). A recent meta-analysis has reported little effect of *S. cerevisiae* supplementation on the DM intake (Abdelli et al., 2022), although others have reported an increase (Desnoyers et al., 2009).

In contrast, the majority of studies report an increase in milk yield with *S. cerevisiae* supplementation (Abdelli et al., 2022; Desnoyers et al., 2009). There is, however, considerable variation between individual studies, depending on the interaction between the supplement and the forage/concentrate ratio of the TMR (Desnoyers et al., 2009). In the current study, the lack of effect on the milk yield and composition could have been due to the relatively low concentrate proportion or the lack of statistical power.

### Rumen metabolism

In the current study, the rumen pH rapidly declined post-feedout regardless of CA and YS. The decline in rumen pH post-feeding has been well documented (Tayyab et al., 2019) and reflects the greater activity of the rumen microbes with increased availability of fermentable carbohydrates (Zhang et al., 2020). The effect of CA was only found as part of an interaction with sampling time, indicating that the ingestion of large amounts of concentrates typical of a poorly mixed TMR will lead to a more rapid increase in rumen fermentation, stabilising during the course of the day. In the current study, the mean minimum pH did not fall below 5.8 for all sampling times. However, in susceptible animals and with higher concentrate proportions, low pH in the rumen could lead to the potential risk of SARA, which is diagnosed as ruminal pH of 5.8 or below for over 5–6 h per day (Zebeli et al., 2012). Thus, in the present study, the addition of YS did not increase the rumen pH sufficiently to be biologically relevant. However, the effect of increased doses of YS and their interactions with higher proportions of concentrates would add to the knowledge of YS and its benefits in different nutritional models.

The influence of yeast and yeast product supplementation on ruminal pH is variable (Baker et al., 2022). However, the majority of studies support the view that a reduction in ruminal lactate concentration and the greater microbial diversity often associated with feeding yeast can lead to an increase in ruminal pH (Desnoyers et al., 2009). The ruminal lactate concentrations in the current study were, however, low and undetectable (<1 mM) in many of the samples, although the propionate concentrations were higher, indicating increased microbial activity. Previous studies have reported either no effect on the ruminal VFA concentration when live yeast was supplemented (Johnson et al., 2023) or an increase in the concentration (Desnoyers et al., 2009; Baker et al., 2022). However, the reported effects were often small and factors such as the concentrate feeding level and the yeast type and dose can all have an influence (Baker et al., 2022).

### Rumen microbiome

The primary objective of the study was to determine the dietary effects on the rumen microbiome. The phylum composition of the microbiome libraries was consistent with a typical rumen microbial community (Henderson et al., 2015), with the bacterial features assigned to Bacteroidota (Bacteroidetes), Bacillota (Firmicutes), and Pseudomonadota (Proteobacteria) along with members of the methanogenic Archaea making up the majority of the dataset. The presence of some of the low-abundance phyla, e.g., Actinobacteria or Cyanobacteria, which are associated more with free-living habitats than the gut, has been previously reported in rumen digesta samples (Suľák et al., 2012). It is possible that this may be a result of ingestion directly from the environment or the contamination of feed or water.

Bray–Curtis dissimilarity between the rumen microbiome was detected with YS, which was associated with a tendency for a higher rumen pH, higher total VFA and propionate concentrations, and a tendency for a higher acetate concentration. This was consistent with similar effects of YS on rumen metabolism and microbiome reported in previous studies (Chaucheyras-Durand et al., 2016; Pinloche et al., 2013).

ANCOM-BC and LEfSe were used to determine discriminant features as both are considered effective and equally robust tools, but differ in their statistical philosophy. Each tool provided different results, which highlights the difficulty of making firm conclusions from the analysis of the microbiome data. Moreover, many of the strains identified using molecular methods have not been isolated, making identification challenging.

In the present study, ANCOM-BC detected discriminant features only in association with YS. Taxonomic classification to the nearest related type strain identified two features with higher abundance and five features with decreased abundance. The discriminant features were all either Bacteroidota or Bacillota, with one feature classified as a member of the Spirochaetota phylum.

Some of the type strains identified were part of a bulk deposition of strains to culture collections. For example, *Ruminococcoides intestinale* is one of 340 strains deposited as part of the Human intestinal Bacteria Collection (HiBC) (Hitch et al., 2024). Others such as *Treponema porcinum* and *Sangeribacter muris* were isolated from the gut of a pig and a mouse, respectively (Nordhoff et al., 2005; Forster et al., 2022). However, the percentage identity between the feature sequence and the type strain sequence was, in the majority of cases, quite low, with *T. porcinum* and *R. intestinale* at 95.5% and with the remainder at less than 90%. This limited the accuracy of the classification to approximately the genus level or above for the former and to the family level or above for the remainder (Edgar, 2018). The lack of closely related cultured strains further highlights the lack of knowledge of the species contained in the rumen microbiome despite efforts to expand the collection using both microbiological (Seshadri et al., 2018) and computational methods (Stewart et al., 2018).

‘Oxygen scavenging’ by yeast has been suggested as a mechanism to increase rumen pH, thus providing favourable conditions for the growth of fibre-degrading bacteria (Chaucheyras-Durand et al., 2016; Pinloche et al., 2013). This is supported by the LEfSe, which identified the family Christensenellaceae associated with YS that has an important role in the metabolism of dietary fibre and has previously been shown to benefit from diets with YS (Chaucheyras-Durand et al., 2016; Newbold et al., 1996). Members of the family Christensenellaceae are also indicators of a healthy metabolic profile in the human gut (Waters and Ley, 2019). The feature identified here was distantly related to the type strain *Gracilibacter thermotolerans* (87%) and thus is a novel species of Gammaproteobacteria yet to be isolated from the rumen. Gammaproteobacteria species have been previously detected in the rumen microbiome and have been proposed as members of the succinate-producing family Succinivibrionaceae (Johnson et al., 2023; Snelling et al., 2019). Increased succinate in the human gut has been reported to play a role in intestinal inflammation (Macias-Ceja et al., 2019), and although succinate was not found in detectable concentrations in the current study (<1 Mm), there was an increase in propionate supplementation with YS. This may have promoted the activity of the Acidaminococcaceae species, which are abundant in the rumen and are capable of metabolising succinate to propionate when it accumulates following ingestion of high quantities of rapidly fermentable dietary concentrates (Marounek et al., 1989).

### Study scope and limitations

Samples taken via rumen cannula comprise the gold standard for analysis of the rumen microbial community and fermentation products. However, it is acknowledged that this design and the number of animals used in the current study may have limited its statistical power. *A priori* power calculations were undertaken based on the predicted treatment differences in rumen fermentation and the microbiome, as this was the primary objective, with other recent studies also using a similar design (Luan et al., 2024; Tayyab et al., 2022). In the present study, the *β* error probability at 0.60 was lower than the preferred value (0.80), and it was recognised that this would lead to an increased probability of type II errors in the production and rumen metabolism data.

## Conclusions

Based on the interaction between CA and sample time, CA only affected the diurnal fluctuation of rumen pH and specifically the rapid drop in rumen pH immediately post-feedout. The effect of CA on the mean rumen pH was not significant. CA had little effect on the microbiome, with Prevotellaceae as the only associated taxonomic biomarker. Supplementation of the diet with live yeast decreased the abundance of features classified as putative Succinivibrionaceae and Prevotellaceae, with an increase in the abundance of an unclassified species of Christensenellaceae, the latter involved with the metabolism of dietary fibre. Yeast supplementation also tended to increase the rumen pH and promote microbial activity with increased VFA production.

## Data Availability

The datasets presented in this study can be found in online repositories. The names of the repository/repositories and accession number(s) can be found below: https://www.ebi.ac.uk/ena/browser/view/PRJEB44109.

## Supplementary material

Supplementary Table 1 Nutritional composition and fermentation characteristics (g/kg DM) of grass silage (GS), maize silage (MS), concentrates (without (–), or with yeast supplementation (+)) and partial mixed ration (PMR; without (–) or with yeast supplementation (+)). ^1^Concentrate composition (g/kg): wheat 320; soyhulls 178; sugarbeet pulp (dried and molassed) 130; soybean meal 170; rapeseed meal 100; molasses (cane) 60; rumen protected fat 8; limestone 16.5; sodium chloride 10; calcined magnesite 5; minerals and vitamins 2.5.
