# Correction: Modulation of the rumen microbiome and metabolism in dairy cows by altering the concentrate feeding pattern and the inclusion of *Saccharomyces cerevisiae* yeast

**DOI:** 10.3389/frmbi.2026.1965017

**Published:** 2026-09-18

**Authors:** Timothy J. Snelling, Catherine A. Johnson, Helen E. Warren, Jules Taylor-Pickard, James A. Huntington, Liam A. Sinclair

**Affiliations:** 1 Animal Science Research Centre, Harper Adams University, Edgmond, United Kingdom; 2 Alltech Bioscience Centre, Dunboyne, Ireland

**Keywords:** concentrates, diet selection, microbiome, pH, rumen, volatile fatty acids

In the original publication, we misstated the amount of Yea Sacc included in cows’ diet – we said that the manufacturer’s recommended rate was 10 g per cow per day, when this should have been 1 g per cow per day.

A correction has been made to the **Abstract**. The sentences, “The 4 × 4 Latin square design consisted of four dairy cows fitted with permanent rumen cannulae, which were fed a partial mixed ration with dietary concentrates (4 kg per cow per day) in an even or an uneven pattern of allocation (concentrate allocation, CA). YS was included in the TMR at a rate of 10 g per cow per day”, have been updated to read:

“The 4 × 4 Latin square design consisted of four dairy cows fitted with permanent rumen cannulae, which were fed a partial mixed ration with dietary concentrates (4 kg per cow per day) in an even or an uneven pattern of allocation (concentrate allocation, CA). YS was included in the TMR at a rate of 1 g per cow per day”

A correction has been made to the section **Materials and methods**, subsection *Animals, diets and management*, paragraph one. The sentence, “The concentrates were prepared with (+) or without (−) live *S. cerevisiae* yeast (YS) (Yea Sacc®, Alltech, Nicholasville, KY, USA; contents: 1 × 10^9^ colony forming units per gram product) to provide the manufacturer’s recommended rate of 10 g per cow per day” has been updated to read:

“The concentrates were prepared with (+) or without (−) live *S. cerevisiae* yeast (YS) (Yea Sacc®, Alltech, Nicholasville, KY, USA; contents: 1 × 10^9^ colony forming units per gram product) to provide the manufacturer’s recommended rate of 1 g per cow per day.”

A correction has been made to the section **Discussion**, subsection *Animal performance*, paragraph one. This originally read: “In high-yielding dairy production systems, effective mixing of the TMR is essential to provide a steady supply of nutrients to support production and body condition and to maintain digestive health (Schingoethe, 2017; Tayyab et al., 2018). In the present study, the effect of poor diet mixing and diet selection was simulated by providing different patterns of CA relative to the time of feedout. It is recognised that this approach does not fully replicate the effect of diet selection on-farm as cows were fed individually and the daily concentrate allowance was kept constant across treatments in the current study. In contrast, diet selection under commercial conditions results not only in a greater intake rate of rapidly fermentable concentrates post-feeding but also in a proportion of the cows consuming a greater amount of concentrates, which can impact the rumen microbiome, increase the risk of SARA, and affect animal performance (Cheng et al., 2023; Tayyab et al., 2022; Zhang et al., 2020).” This has been updated to read:

“In high-yielding dairy production systems effective mixing of the TMR is essential to provide a steady supply of nutrients to support production and body condition and to maintain digestive health (Schingoethe, 2017; Tayyab et al., 2018). In the present study, the effect of poor diet mixing and diet selection was simulated by providing different patterns of concentrate allocation relative to time of feed out. It is recognised that this approach does not fully replicate the effect of diet selection on-farm, as cows were fed individually and the daily concentrate allowance kept constant across treatments in the current study. In contrast, diet selection under commercial conditions results not only in a greater rate of intake of rapidly fermentable concentrates post-feeding, but also in a proportion of the cows consuming a greater amount of concentrates, which can impact the rumen microbiome, increase risk of SARA, and affect animal performance (Cheng et al., 2023; Tayyab et al., 2022; Zhang et al., 2020). *Saccharomyces cerevisiae* yeast’s (YS) mode of action (MOA) is described as oxygen scavenging: production of cofactors (small peptides) that stimulate bacterial growth and sugar-scavenging. Here, the MOA was considered to be directed toward metabolic activity and oxygen scavenging, but replication was considered less of an impact; hence, the additive was fed on a daily basis according to manufacturer’s recommendations.”

The original version of this article has been updated.

