# Supplementary material for: Modulation of the rumen microbiome and metabolism in dairy cows by altering the concentrate feeding pattern and the inclusion of Saccharomyces cerevisiae yeast
Source: Front Microbiomes. 2026 Aug 7;5:1884444. doi: 10.3389/frmbi.2026.1884444 (PMC13493597; doi:10.3389/frmbi.2026.1884444)
Supplement: Supplementary file 1 [file DataSheet1.docx]

| Supplementary Table 1. Nutritional composition and fermentation characteristics (g/kg DM) of grass silage (GS), maize silage (MS), concentrates (without (–), or with yeast supplementation (+)) and partial mixed ration (PMR; without (–) or with yeast supplementation (+)) | | | | | | |
| --- | --- | --- | --- | --- | --- | --- |
|  | **GS** | **MS** | Concentrates (–)^1^ | Concentrates (+)^1^ | PMR (-) | PMR (+) |
| DM | 233 | 356 | 883 | 879 | 323 | 316 |
| CP | 183 | 80.3 | 198 | 209 | 149 | 150 |
| Ash | 129 | 34.0 | 78.0 | 67.0 | 92 | 90 |
| OM | 845 | 966 | 922 | 933 | 908 | 910 |
| NDF | 441 | 373 | 236 | 249 | 423 | 402 |
| *Fermentation characteristics* | | | | | | |
| pH | 4.19 | 4.37 |  |  |  |  |
| NH_3_-N, g/kg of total N | 27.1 | 23.6 |  |  |  |  |
| Acetate | 42.6 | 43.5 |  |  |  |  |
| Propionate | 1.13 | 1.47 |  |  |  |  |
| Iso-butyrate | 0.1 | 0.16 |  |  |  |  |
| Butyrate | 1.99 | 2.10 |  |  |  |  |
| Iso-valerate | 0.17 | 0.23 |  |  |  |  |
| Lactate | 82.4 | 67.6 |  |  |  |  |
| ^1^Concentrate composition (g/kg): wheat 320; soyhulls 178; sugarbeet pulp (dried and molassed) 130; soybean meal 170; rapeseed meal 100; molasses (cane) 60; rumen protected fat 8; limestone 16.5; sodium chloride 10; calcined magnesite 5; minerals and vitamins 2.5. | | | | | | |
